# Progressive Dysphagia in Patient With Cervical Plate Complicated With Posterior Pharyngeal Wall Erosion

**DOI:** 10.7759/cureus.25205

**Published:** 2022-05-22

**Authors:** Davood K Hosseini, Ramtin Moradi, Tyler Schoch, Lesley Philip, Nilesh B Shukla

**Affiliations:** 1 Internal Medicine, Hackensack University Medical Center, Hackensack, USA; 2 Internal Medicine, Richmond University Medical Center, Staten Island, USA; 3 Medicine, Hackensack University Medical Center, Hackensack, USA; 4 Gastroenterology, Hackensack University Medical Center, Hackensack, USA

**Keywords:** esophagogastroduodenoscopy (egd), mri- magnetic resonance imaging, cervical fixation plate, pharyngeal wall erosion, progressive dysphagia

## Abstract

A 58-year-old male patient with a history of Parkinson's disease and solitary cervical spinal sarcoma underwent corpectomy, a fusion of C3-C6 with cervical fixation plate placement, and stereotactic body radiation therapy, presented 18 months following surgery with dysphagia, concomitant with weakness, diplopia. The initial workup in cervical magnetic resonance imaging (MRI) revealed aerodigestive tract soft tissue enhancement. Dysphagia progressed during hospitalization, and the patient was intubated due to aspiration pneumonia and respiratory failure. Further evaluations with esophagogastroduodenoscopy (EGD) revealed posterior pharyngeal wall, upper cervical esophageal erosion, and the presence of a cervical fixation plate in the hypopharynx.

## Introduction

Esophageal erosion following anterior cervical spine surgery is rare and reported to be between 0.02 and 1.49%, and it has a mortality rate close to 6 percent [1]. Although most esophageal erosions occur intra-operative or immediately following surgical intervention, few cases have been reported with a delayed presentation [2]. Diagnosis of esophageal perforation can be made with cervical imaging studies, including X-ray, computed tomography (CT) scan, and magnetic resonance imaging (MRI). However, negative imaging does not rule out esophageal injury, and further evaluation with surgical exploration is warranted in the presence of high clinical suspicion.

## Case presentation

**Figure 1:**
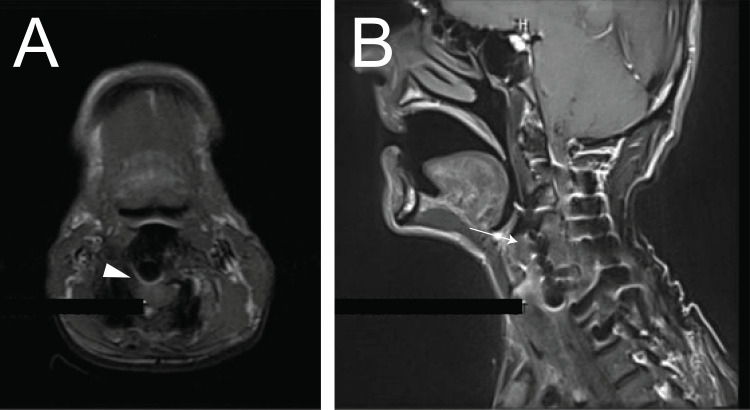
Neck MRI. The absence of expected soft tissue with the posterior wall is suggestive of hardware erosion (arrowhead- 1A). There is an abnormal thickening of the laryngeal soft tissues, with medialization of the right vocal cord and loss of expected parapharyngeal fat (arrow- 1B).

A 58-year-old male patient with a past medical history significant for Parkinson's disease and solitary cervical spinal sarcoma underwent corpectomy, a fusion of C3- C6 with cervical fixation plate placement, and stereotactic body radiation therapy and presented with three weeks history of dysphagia, concomitant with weakness, diplopia. His symptoms started eighteen months post-operatively. On presentation, the patient was febrile (Temperature of 103F), with a blood pressure of 112/65 mmHg, heart rate of 98 beats/min, and respiratory rate of 16 per minute. Initial workup revealed leukocytosis (WBC: 11500), with normal Chest X-ray and urine analysis. Further workup was negative for Myasthenia Gravis (Acetylcholine receptor binding antibody less than 0.3 nmol/L). The cervical magnetic resonance imaging (MRI) showed the presence of a metallic cervical plate, and the absence of expected soft tissue with the posterior wall, suggestive of hardware, without evidence of fluid collection or spinal cord compression (Figure 1). However, the evaluation was limited due to magnetic susceptibility artifacts from fusion hardware.

**Figure 2:**
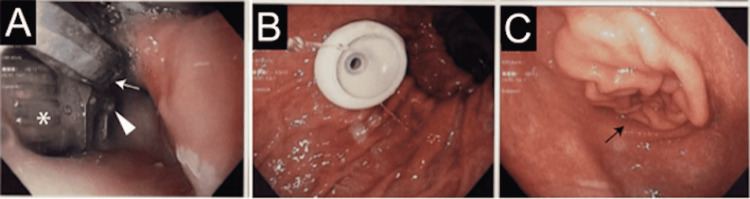
EGD, and PEG placement. Posterior pharyngeal wall and upper cervical esophageal erosion with the presence of cervical fixation plate (white arrow), screws (white arrowhead), and corpectomy fusion cage (white asterisk) in the hypopharynx (Figure 2A). PEG tube placement (Figure 2B) and pyloric valve (black arrow, Figure 2C). PEG: percutaneous endoscopic gastrostomy; EGD: Esophagogastroduodenoscopy

Dysphagia progressed during hospitalization and got complicated with an episode of aspiration pneumonia during ingestion of medication, which progressed to respiratory failure requiring intubation and mechanical ventilation. The patient received empirical Piperacillin-Tazobactam while the sputum culture was positive for Pseudomonas aeruginosa (while the blood culture was negative). The patient subsequently underwent endoscopic gastroesophageal duodenoscopy (EGD) for further evaluation and percutaneous endoscopic gastrostomy (PEG) placement in the body of the stomach (due to dysphagia and complicated aspiration pneumonia). EGD revealed erosion of the posterior pharyngeal wall and upper cervical esophagus and the presence of a cervical fixation plate, screws, and corpectomy fusion cage in the hypopharynx (Figure 2). 

**Figure 3:**
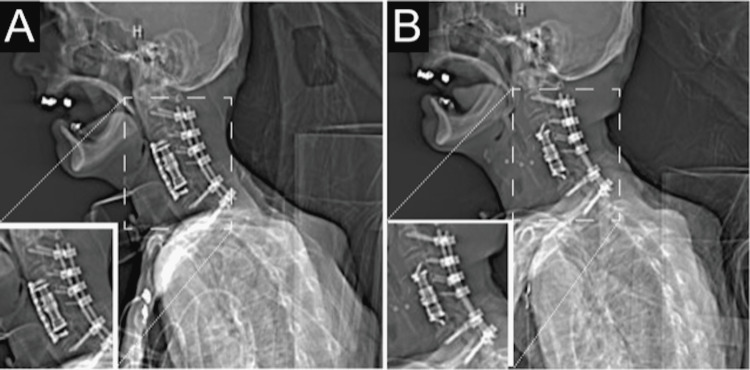
CT neck with contrast. Posterior fusion device with a facet and pedicle screws at C2-C5, C7, and T1 with anterior fusion plate and screw construct extending from the C3 level through the C6 level (Figure 3A). Post-surgical removal of the anterior fusion plate and screws (Figure 3B).

The orthopedic surgery and otolaryngology-head and neck surgery services were consulted. The patient underwent surgical exploration of the cervical spine. The anterior cervical fixation plate was removed with flap reconstruction, and the cervical dural tear was repaired with a resolution of his symptoms (Figure 3). The patient was discharged to a rehabilitation facility.

## Discussion

The esophagus lies directly anterior to the cervical spine, and it is vulnerable to injury post-operatively. Adventitia, the outermost esophageal layer, protects the underneath layers, including the muscular layer (longitudinal and circular) and the submucosal and mucosal layers.

During anterior cervical surgery, aggressive or improper retraction of the esophagus may lead to esophageal erosion, a challenging clinical problem. Although most esophageal injuries occur intra-operative or immediately following surgical intervention, few cases are reported with a delayed presentation [2,3]. Symptoms include dysphagia and Mackler's triad (subcutaneous emphysema, chest pain, and vomiting) in the setting of esophageal perforation [4].

Early diagnosis and intervention reduce morbidity and mortality, so any intraoperative suspicion should warrant immediate investigation. Diagnosis usually requires direct visualization or imaging studies, including endoscopy, CT-scan, MRI, or contrast swallow studies [5,6]. Treatment modalities include non-surgical, conservative management, and primary closure with flap placement [3]. Brinster et al. reported that interval time from perforation to repair of less than 24 hours had been associated with a significant reduction in morbidity and mortality [7].

Surgical outcomes also depend on the pre-operative comorbidity. Bhatia et al. reported that pulmonary comorbidity, development of sepsis, and respiratory failure requiring mechanical ventilation at presentation significantly impact overall outcome [8]. They also reported that the site of the esophageal perforation is an essential factor determining the severity of the disease, with the cervical esophageal perforations tend to incite less of a systemic inflammatory response than thoracic and abdomen perforation [8].

## Conclusions

Esophageal injuries following anterior cervical spine surgery are a potential and rare complication reported in the literature, usually detected during or acutely following surgery. Our patient presented with progressive dysphagia 18 months after anterior cervical surgery. Interestingly, he was asymptomatic for months following the surgery, and dysphagia was the initial complaint that warranted further evaluations. High clinical suspicion is required to detect esophageal injuries and warrant early intervention and correction.
